# Increased Risk for Cerebral Hypoxia During Immediate Neonatal Transition After Birth in Term Neonates Delivered by Caesarean Section With Prenatal Tobacco Exposure

**DOI:** 10.3389/fped.2021.747509

**Published:** 2021-11-23

**Authors:** Christina Helene Wolfsberger, Marlies Bruckner, Bernhard Schwaberger, Lukas Peter Mileder, Ena Pritisanac, Nina Hoeller, Alexander Avian, Berndt Urlesberger, Gerhard Pichler

**Affiliations:** ^1^Division of Neonatology, Department of Pediatrics and Adolescent Medicine, Medical University of Graz, Graz, Austria; ^2^Institute for Medical Informatics, Statistics and Documentation, Medical University of Graz, Graz, Austria

**Keywords:** term neonates, immediate neonatal resuscitation, near infrared spectroscopy, NIRS, cerebral oxygenation, maternal smoking, prenatal tobacco exposure, nicotine

## Abstract

**Introduction:** Maternal tobacco smoking during pregnancy is a global health problem leading to an increased risk for fetal and neonatal morbidities. So far, there are no data of the potential impact of maternal smoking during pregnancy on the most vulnerable period after birth – the immediate postnatal transition. The aim of the present study was therefore, to compare cerebral oxygenation during immediate postnatal transition in term neonates with and without prenatal tobacco exposure.

**Methods:** Included in this *post-hoc* analysis were healthy term neonates, with measurements of cerebral oxygenation (INVOS 5100C) during the first 15 min after birth, and for whom information on maternal smoking behavior during pregnancy was available. Neonates with prenatal tobacco exposure (smoking group) were matched 1:1 according to gestational age (±1 week), birth weight (±100 grams) and hematocrit (±5 %) to neonates without (non-smoking group). Cerebral regional tissue oxygen saturation (crSO_2_), cerebral fractional tissue oxygen extraction (cFTOE), arterial oxygen saturation (SpO_2_) and heart rate (HR) within the first 15 min after birth were compared between the two groups.

**Results:** Twelve neonates in the smoking group with a median (IQR) gestational age of 39.1 (38.8–39.3) weeks and a birth weight of 3,155 (2,970–3,472) grams were compared to 12 neonates in the non-smoking group with 39.1 (38.7–39.2) weeks and 3,134 (2,963–3,465) grams. In the smoking group, crSO_2_ was significantly lower and cFTOE significantly higher until min 5 after birth. HR was significantly higher in the smoking group in min 3 after birth. Beyond this period, there were no significant differences between the two groups.

**Conclusion:** Cerebral oxygenation within the first 5 min after birth was compromised in neonates with prenatal tobacco exposure. This observation suggests a higher risk for cerebral hypoxia immediately after birth due to fetal tobacco exposure.

## Introduction

Tobacco smoking during pregnancy is a global health problem with a prevalence of 1.7%, whereby the highest prevalence of 8.1% is seen in Europe. Regrettably, 30.6% of European women, who smoke daily before pregnancy, continue smoking during pregnancy (1).

Maternal tobacco smoking during pregnancy is significantly linked to fetal and neonatal morbidities and mortality. Pregnancy related complications including placental abruption or preeclampsia (2) result from impaired placental and umbilical circulation, inducing increased risk for neonatal adverse outcome (3). Prenatal tobacco exposure is further related to preterm birth (4), lower birth weight, smaller head circumference, fetal growth restriction, neurological morbidities, major congenital malformations, and sudden infant death syndrome (5–11).

Effects of prenatal tobacco exposure on neonatal cerebral oxygenation and perfusion after birth can be assessed by near-infrared spectroscopy (NIRS), which enables continuous and non-invasive monitoring of cerebral regional tissue oxygen saturation (crSO2) and cerebral fractional tissue oxygen extraction (cFTOE) by measuring oxygenated (HbO2) and deoxygenated hemoglobin (Hb) (12–15). When near-infrared light propagates through tissues, it is differently absorbed by HbO2 and Hb. Relative changes in HbO2 and Hb in a tissue can be calculated out of changes in the attenuation of light (16, 17). In an observational study in preterm neonates, who developed cerebral injury during the first week after birth, higher burden of cerebral hypoxia during the first 15 min after birth has already been observed (18). Measurement of cerebral oxygenation with NIRS during immediate postnatal transition in the delivery room is meanwhile well-established (19–24) and several factors influencing cerebral oxygenation within the first 15 min after birth have already been identified (25–31).

Prenatal tobacco exposure influences cerebral oxygenation during the first 2 days after birth in preterm neonates, who show lower crSO2 and higher cFTOE compared to neonates without prenatal tobacco exposure (32). A prospective cohort study has demonstrated a significant difference in peripheral (muscle) tissue oxygenation within the first 48 h after birth in healthy term neonates with prenatal tobacco exposure compared to neonates without (33). In this study, peripheral muscle tissue oxygenation was significantly lower and peripheral muscle fractional oxygen extraction was significantly higher in the smoking group within the first 24 h after birth.

However, so far no study has investigated the potential influence of maternal smoking during pregnancy on neonates during the most vulnerable period after birth—the immediate postnatal transition.

The aim of the present study was to evaluate the potential influence of prenatal tobacco exposure on cerebral oxygenation in clinically stable term neonates during the immediate neonatal transition after birth. Therefore, cerebral oxygenation of neonates from mothers who had smoked during pregnancy were compared to those from mothers who had not smoked during pregnancy. Based on existing studies, we hypothesized that cerebral oxygenation in neonates from mothers who had smoked during pregnancy would be compromised.

## Materials and Methods

### Design

For the present study a *post-hoc* analysis of secondary outcome parameters of a prospective observational study (34), which was conducted at the Division of Neonatology, Department of Pediatrics and Adolescent Medicine, Medical University of Graz, Austria, between October 2015 and June 2018, was performed. Written parental consent had been obtained prior to birth and inclusion in the observational study. The study had been approved by the Regional Committee on Biomedical Research Ethics (EC numbers: 27–465 ex 14/15) and was carried out in accordance with The Code of Ethics of the World Medical Association (Declaration of Helsinki) (35).

### Inclusion and Exclusion Criteria

Included in the present *post-hoc* analysis were term neonates, delivered by Cesarean section, with available maternal information on smoking behavior during pregnancy, including smoking yes/no and number of smoked cigarettes per day. We included only term neonates with NIRS measurements during the first 15 min after birth and with a blood gas analysis performed within the first 25 min after birth, to obtain the hematocrit value.

Exclusion criteria included major congenital anomalies as well as the need for any kind of medical support [medications, supplementary oxygen and/or respiratory support including continuous positive airway pressure (CPAP) or intubation], to eliminate any influence on cerebral oxygenation.

### Groups

The neonates were stratified into two groups according to maternal smoking during pregnancy: neonates with maternal smoking were assigned to the “smoking group,” and neonates from mothers who had not smoked during pregnancy to the “non-smoking group.” Neonates in the smoking group were matched for gestational age ± 1 week and birth weight ± 100 g to healthy term neonates in the non-smoking group. In addition, groups were matched for hematocrit ± 5% to rule out a potential influence of hemoglobin on cerebral oxygenation.

### Monitoring and Postnatal Management

Information on maternal smoking behavior during pregnancy including number of daily smoked cigarettes, maternal characteristics, antepartum medical history, and neonatal demographic data including gestational age, birth weight, sex, umbilical artery pH, Apgar scores and results of the first, routinely performed blood gas analysis were documented.

The neonates were delivered by planed Cesarean section, without labor and cord clamping was routinely delayed for 30 s after birth. Immediately after the cord was clamped the neonates were placed under an overhead heater in supine position dried and wrapped in warm towels.

NIRS measurements were performed immediately after birth under standardized conditions. The forehead was cleaned to remove vernix and amniotic fluid, as these could affect probe contact. The NIRS sensor was placed on the neonates' left fronto-parietal head and was fixed with a CPAP cap or an elastic bandage. NIRS measurements were continuously performed during the first 15 min after birth with an INVOS 5100C Cerebral/Somatic Oximeter Monitor (Medtronic, Minneapolis, MN, U.S.A.) and a neonatal transducer. cFTOE was calculated out of crSO_2_ and arterial oxygen saturation (SpO_2_) using the following formula: cFTOE = (SpO_2_-crSO_2_)/SpO_2_ (36). According to a quality criterion for cerebral measurements, crSO_2_ and SpO_2_ values were eliminated when crSO_2_ was higher than SpO_2_ (37).

SpO_2_ and heart rate (HR) were measured with pulse oximetry, applied on the neonates' right hand or wrist, using the IntelliVue MP30 monitor (Koninklijke Philips, Amsterdam, The Netherlands).

All variables were recorded continuously during the first 15 min after birth and were stored in a multichannel system (alpha-trace digital MM, B.E.S.T. Medical Systems, Vienna, Austria) for subsequent analyses. Values of crSO_2_ SpO_2_ and HR were stored every second.

Using a pneumatic cuff on the neonates' right upper arm, non-invasive blood pressure (MABP) was measured once in minute 15 after birth. The IntelliVue MP50 monitor (Koninklijke Philips, Amsterdam, The Netherlands) was used for this purpose. Rectal body temperature was also measured once in minute 15 after birth.

### Blood Gas Analysis

Hematocrit, hemoglobin and fetal hemoglobin (HbF) values as well as pH, bicarbonate, base excess, partial oxygen pressure (pO_2_) and partial carbon dioxide pressure (pCO_2_) were measured by blood gas analysis from a capillary sample within 25 min after birth. Samples were obtained by a heel-stick only at the discretion of the attending neonatologist. The analysis was performed according to local standard operating procedures with a blood gas analyzer (ABL 800 Flex, Drott, Wiener Neustadt, Austria).

### Statistical Analysis

Data are presented as mean and standard deviation (SD) or median and interquartile range (IQR) for continuous data and absolute and relative frequency for categorical data, respectively. Baseline differences between groups were analyzed using *t*-test or Mann–Whitney *U*-test for and Chi-square test, as appropriate. A linear mixed model with fixed effects for time and nicotine use and interaction of time and nicotine use applying a first-order autoregressive covariance structure was used for calculation of overall effects and differences between groups at each minute. Course of parameters were analyzed starting with the third minute after birth until minute 15. The first 2 min of life were not analyzed due to the high number of missing values. For the visualization of the courses of analyzed parameters, estimated values according to the linear mixed model with 95% confidence intervals (95% CI) are shown. A *p*-value of *p* < 0.05 was considered statistically significant. Statistical analyses were performed using SPSS 26.0 (SPSS, Chicago, IL, USA).

## Results

**Figure 1:**
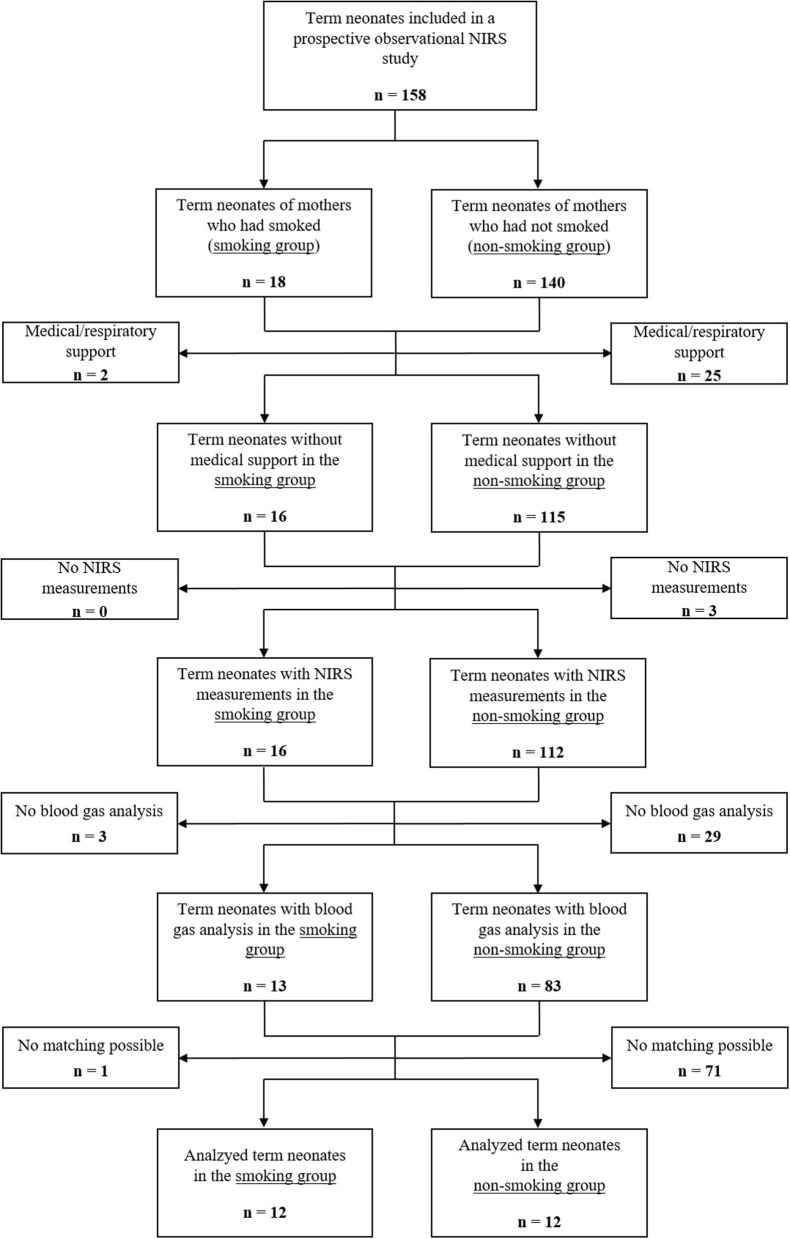
Study flow chart showing the number of included term neonates and rationales for exclusion.

Eligible were 158 term neonates, who were included in the prospective observational NIRS study. Eighteen neonates were stratified to the smoking group and 140 to the non-smoking group. Two neonates in the smoking group and 25 neonates in the non-smoking group were excluded due to the need for medical support, including supplementary oxygen and/or respiratory support. None of the neonates in the smoking group and three neonates in the non-smoking group were excluded as no NIRS measurements were available within the first 15 min after birth. Three neonates in the smoking group and 29 neonates in the non-smoking group had no blood gas analysis performed within the first 25 min after birth and were therefore excluded. Furthermore, one neonate in the smoking group and 71 neonates in the non-smoking group were excluded, as no matching according to gestational age, birth weight and hematocrit was possible. Finally, 12 neonates in the smoking group and 12 neonates in the non-smoking group fulfilled inclusion and matching criteria (Figure 1).

There were no differences in maternal characteristics including maternal age at the time of delivery [31.0 (27.2–35.3) years in the smoking group and 31.0 (26.6–34.2) years in the non-smoking group; *p* = 0.743] and parturition [2.0 (1.6–2.8) in the smoking group and 1.0 (1.1–2.1) in the non-smoking group; *p* = 0.114]. Mothers in the smoking group had a body-mass index of 31.8 ± 8.4 and in the non-smoking group 28.3 ± 6.0; *p* = 0.387. In both groups, there were no severe preexisting maternal morbidities. Except for medication treating hypothyroidism (smoking group *n* = 2; non-smoking group *n* = 4), the mothers had no additional medications. The mothers in the smoking group reported to smoke median (IQR) 8 (4–10) cigarettes per day.

Indications for the Cesarean sections were previous Cesarean section (*n* = 7), breech position (*n* = 3), and maternal request (*n* = 2) in the smoking group and previous Cesarean section (*n* = 6), breech position (*n* = 3), maternal request (*n* = 2), and preeclampsia (*n* = 1) in the non-smoking group. Anesthesia during Cesarean section was in all cases epidural anesthesia, except of one mother in the smoking group, who received general anesthesia.

Neonatal characteristics and results of capillary blood samples are presented in Table 1. Sampling time for blood gas analyses was in median (IQR) in minute 16.0 (15.0–16.5) in the smoking group and 16.5 (15.0–18.0) in the non-smoking group.

**Table 1:** Characteristics of term neonates from mothers who had smoked during pregnancy and in term neonates from mothers who had not smoked during pregnancy (smoking group and non-smoking group).

	**Smoking** **(*n* = 12)**	**Non-smoking** **(*n* = 12)**	***p*-value**
**Demographic data**			
Gestational age, median (IQR), weeks	39.1 (38.8–39.3)	39.1 (38.7–39.2)	0.502
Birth weight, mean ± SD, *g*	3,165 ± 341	3,153 ± 330	0.927
Female sex, *n* (%)	6 (50)	6 (50)	1.000
Umbilical artery pH, median (IQR)	7.32 (7.31–7.34)	7.33 (7.32–7.34)	0.920
Apgar 1 min, median (IQR)	9 (9)	9 (9)	0.317
Apgar 5 min, median (IQR)	10 (10)	10 (10)	0.623
Apgar 10 min, median (IQR)	10 (10)	10 (10)	0.317
**Routine Monitoring**			
Mean arterial blood pressure, mean ± SD, *mmHg*	51 ± 12	53 ± 11	0.719
Rectal body temperature, mean 0 ± SD, *°C*	37.2 ± 0.4	37.2 ± 0.3	0.954
**Blood gas analysis**			
Hematocrit, mean ± SD, %	59.6 ± 5.4	58.7 ± 3.7	0.660
Hemoglobin, mean ± SD, *g/dl*	19.5 ± 1.8	19.2 ± 1.3	0.626
HbF, mean ± SD, %	69.7 ± 10.7	74.08 ±5.4	0.217
pH, mean ± SD	7.30 ± 0.04	7.30 ± 0.04	0.861
Bicarbonat, mean ± SD, *mmol/l*	21.82 ± 1.35	22.11 ± 1.17	0.578
Base excess, mean ± SD	−0.98 ± 1.37	−0.43 ± 1.28	0.319
pO_2_, mean ± SD, *mmHg*	39.5 ± 5.9	40.4 ± 4.4	0.684
pCO_2_, mean ± SD, *mmHg*	51.7 ± 6.0	53.6 ± 7.5	0.519

*Data are presented as mean ± SD, median (IQR) or n (%). IQR, interquartile range; SD, standard deviation; HbF, fetal hemoglobin; pO_2_, partial pressure of oxygen; pCO_2_, partial pressure of carbon dioxide*.

**Figure 2:**
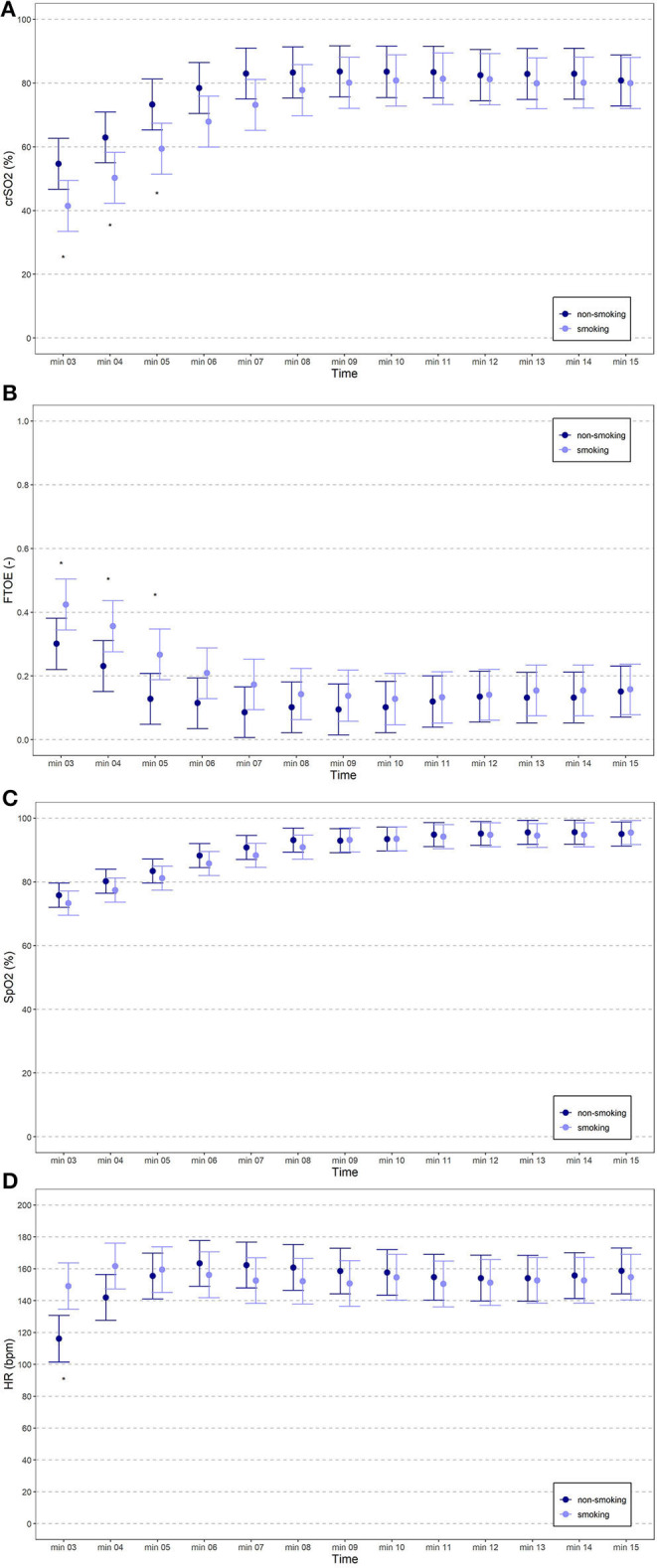
**(A–D)** crSO_2_, cFTOE, SpO_2_ and HR of term neonates from mothers who had smoked during pregnancy (light blue spots) compared to term neonates from mothers who had not smoked during pregnancy (dark blue spots).

The courses of crSO2 and cFTOE during the first 15 min after birth are demonstrated for the two groups in Table 2; Figures 2A,B. crSO2 was significantly lower until min 5 after birth (in both groups: min 3: *n* = 11, min 4: *n* = 12, min 5: *n* = 11) and cFTOE was significantly higher until min 5 after birth (in both groups: min 3: *n* = 10, min 4: *n* = 11, min 5: *n* = 11) in the smoking group compared to the non-smoking group. SpO2 and HR during the first 15 min after birth are demonstrated in Table 3; Figures 2C,D. There were no significant differences between the two groups in SpO2. HR was significantly higher in the smoking group in min 3 after birth compared to the non-smoking group. From min 4 until 15, there were no further significant differences between the two groups.

**Table 2:** Cerebral regional oxygen saturation (%) and cerebral fractional tissue oxygen extraction in term neonates from mothers who had smoked during pregnancy compared to term neonates from mothers who had not smoked during pregnancy (smoking group and non-smoking group).

**Time after birth**	**crSO**_**2**_			**cFTOE**		
	**Smoking**	**Non-smoking**	***p*-value**	**Smoking**	**Non-smoking**	***p-*value**
	**(*n* = 12)**	**(*n* = 12)**		**(*n* = 12)**	**(*n* = 12)**	
3 min	41.4 (33.4–49.5)	54.7 (46.6–62.7)	0.024*	0.42 (0.34–0.50)	0.30 (0.22–0.38)	0.034*
4 min	50.3 (42.3–58.2)	62.9 (55.0–70.9)	0.029*	0.36 (0.28–0.44)	0.23 (0.15–0.31)	0.031*
5 min	59.4 (51.4–67.4)	73.3 (65.3–81.3)	0.018*	0.27 (0.19–0.35)	0.13 (0.05–0.21)	0.017*
6 min	67.9 (59.9–75.9)	78.4 (70.4–86.4)	0.066	0.21 (0.13–0.29)	0.11 (0.04–0.19)	0.098
7 min	73.2 (65.2–81.1)	83.0 (75.0–91.0)	0.086	0.17 (0.09–0.25)	0.09 (0.01–0.17)	0.125
8 min	77.8 (69.8–85.8)	83.3 (75.3–91.3)	0.326	0.14 (0.06–0.22)	0.10 (0.02–0.18)	0.463
9 min	80.1 (72.0–88.1)	83.6 (75.6–91.7)	0.526	0.14 (0.06–0.22)	0.10 (0.02–0.18)	0.447
10 min	80.8 (72.8–88.9)	83.5 (75.5–91.6)	0.633	0.13 (0.05–0.21)	0.10 (0.02–0.18)	0.652
11 min	81.3 (73.2–89.4)	83.4 (75.3–91.5)	0.709	0.13 (0.05–0.21)	0.12 (0.04–0.20)	0.818
12 min	81.2 (73.2–89.2)	82.5 (74.4–90.5)	0.824	0.14 (0.06–0.22)	0.13 (0.05–0.21)	0.913
13 min	79.9 (71.9–87.9)	82.8 (74.9–90.8)	0.602	0.15 (0.07–0.23)	0.13 (0.05–0.21)	0.691
14 min	80.1 (72.1–88.1)	82.9 (74.9–90.9)	0.619	0.15 (0.07–0.23)	0.13 (0.05–0.21)	0.691
15 min	80.0 (72.0–88.0)	80.8 (72.8–88.8)	0.885	0.16 (0.08–0.24)	0.15 (0.07–0.23)	0.907

Data are presented as estimated mean, 95% CI. P-value <0.05 (*) is considered as statistically significant. crSO_2_, cerebral regional oxygen saturation; cFTOE, cerebral fractional tissue oxygen extraction; min, minute.

**Table 3:** Arterial oxygen saturation (%) and heart rate (bpm) in term neonates from mothers who had smoked during pregnancy compared to term neonates from mothers who had not smoked during pregnancy (smoking group and non-smoking group).

**Time after birth**	**SpO**_**2**_			**HR**		
	**Smoking**	**Non-smoking**	***p*-value**	**Smoking**	**Non-smoking**	***p-*value**
	**(*n* = 12)**	**(*n* = 12)**		**(*n* = 12)**	**(*n* = 12)**	
3 min	73.3 (69.5–77.1)	75.8 (72.0–79.6)	0.356	149.1 (134.5–163.7)	116.0 (101.4–130.6)	0.002*
4 min	77.4 (73.6–81.2)	80.2 (76.4–84.0)	0.297	161.7 (147.3–176.0)	142.0 (127.6–156.3)	0.057
5 min	81.1 (77.4–84.9)	83.4 (79.6–87.1)	0.401	159.5 (145.1–173.8)	155.4 (141.1–169.8)	0.691
6 min	85.7 (82.0–89.5)	88.2 (84.4–92.0)	0.356	156.1 (141.8–170.5)	163.3 (149.0–177.7)	0.481
7 min	88.3 (84.5–92.0)	90.8 (87.0–94.6)	0.344	152.6 (138.2–166.9)	162.3 (147.9–176.6)	0.341
8 min	90.8 (87.1–94.6)	93.1 (89.3–96.8)	0.405	152.2 (137.8–166.5)	160.8 (146.4–175.1)	0.399
9 min	93.2 (89.4–96.9)	92.9 (89.1–96.6)	0.911	150.8 (136.4–165.1)	158.6 (144.2–172.9)	0.445
10 min	93.4 (89.7–97.2)	93.4 (89.6–97.2)	0.992	154.6 (140.3–169.0)	157.6 (143.3–172.0)	0.766
11 min	94.1 (90.4–97.9)	94.8 (91.0–98.6)	0.806	150.4 (136.1–164.8)	154.7 (140.3–169.0)	0.678
12 min	94.7 (91.0–98.5)	95.1 (91.4–98.9)	0.879	151.3 (136.9–165.6)	154.1 (139.8–168.5)	0.781
13 min	94.5 (90.7–98.3)	95.5 (91.7–99.3)	0.713	152.7 (138.3–167.0)	154.0 (139.7–168.3)	0.895
14 min	94.7 (91.0–98.5)	95.6 (91.8–99.4)	0.749	152.7 (138.4–167.1)	155.7 (141.3–170.0)	0.771
15 min	95.4 (91.7–99.2)	95.0 (91.2–98.8)	0.865	154.7 (140.3–169.0)	158.6 (144.3–173.0)	0.700

Data are presented as estimated mean, 95% CI. P-value <0.05 (*) is considered as statistically significant. SpO_2_, arterial oxygen saturation; HR, heart rate; min, minute.

## Discussion

To the best of our knowledge, this study is the first investigating a potential influence of maternal smoking during pregnancy on cerebral oxygenation during the immediate neonatal transition after birth in healthy term neonates. We found that cerebral oxygenation within the first 5 min after birth was lower in infants whose mothers had smoked during pregnancy compared to those of the non-smoking group. These results support our hypothesis that maternal smoking during pregnancy is associated with compromised cerebral oxygenation immediately after birth.

In neonates from mothers who had smoked during pregnancy, polyglobulia (38) and higher HbF values (39) have been reported. These factors may influence cerebral oxygenation, as cFTOE correlates negatively with total hemoglobin concentration. In our study, term neonates with prenatal tobacco exposure were therefore matched for hematocrit value. Moreover, there were no differences in HbF between the two groups. This suggests that other mechanisms have to be responsible for the observed differences in cerebral oxygenation during the immediate neonatal transition after birth.

Cerebral oxygenation immediately after birth in those neonates from mothers who had smoked during pregnancy may display the fetal conditions *in utero*. Nicotine moderates the release of catecholamines from adrenal glands and nerve cells, leading to vasoconstriction in placental and umbilical vessels (40, 41). Tobacco smoking also induces morphological changes in the placenta with infarcts and calcifications, again causing utero-placental vasoconstriction (42). This might be associated with placental insufficiency and chronic fetal hypoxia due to compromised oxygen delivery and nutrients supply to the fetus (38). However, as we did not find any differences in SpO2 and pO2 between the two groups, systemic arterial hypoxia in the smoking group can be ruled out as a cause of the impaired cerebral oxygenation in the smoking group.

HR was significantly higher in min 3 and a trend toward higher values in min 4 after birth in the smoking group was observed in the present study. This is in accordance with reports of increased maternal and fetal HR due to tobacco exposure (38, 43). An explanation for the increased HR is the nicotine induced catecholamine release (42). This effect may probably persist in the first minutes after birth and explains the higher HR in the smoking group. The increased HR in the smoking group would suggest increased cardiac output with improved cerebral perfusion and, thus, cerebral oxygenation. However, in the present study we observed lower crSO2 and higher cFTOE in the smoking group, suggesting—in the case of similar oxygen content of the blood (similar hemoglobin level and SpO2/pO2 levels in both groups)—impaired cerebral perfusion due to vasoconstriction and/or impairment of cerebral autoregulatory capacity in the first minutes after birth.

An increase in cerebral blood flow velocity between 20 and 42 h after birth has already been described in newborn infants from mothers who had smoked during pregnancy (32, 44). This caused an impairment in cerebral perfusion due to changes in cerebrovascular resistance, leading to higher cFTOE (32, 44). This increase of cFTOE and decrease of crSO2 was observed by Verhagen et al. (32) in preterm neonates with prenatal tobacco exposure during the first two days as well as on day eight after birth. On the first day after birth, the median crSO2 was 73 vs. 81% and cFTOE was 0.24 vs. 0.15 when comparing a smoking group to a non-smoking group, respectively (32). We can add to this data, as in the present study crSO2 was significantly lower in the first 5 min after birth in the smoking group, with a trend toward a lower cerebral oxygenation up to minute ten. When comparing the maximum differences of crSO2 and cFTOE of the smoking vs. the non-smoking group from Verhagen et al. (32) on the first day after birth and our results at min 3 after birth (crSO2 41.4% smoking vs. 54.7% non-smoking group; cFTOE 0.42 vs. 0.30), our study revealed an even more pronounced difference in cerebral oxygenation.

### Limitations

We have some limitations to acknowledge. First, the number of cigarettes smoked during pregnancy may be underreported, since they were self-reported by the mothers using a questionnaire; moreover, some mothers in the non-smoking group could have neglected smoking during pregnancy. We did not perform measurements of nicotine levels in hair or urine, which would have informed us on the actual tobacco consumption. Second, background factors that might be associated with smoking were not collected/documented and may have had an impact on the reported results. Third, the number of included neonates in this study is small, therefore interpretation of these results has to be performed with caution. However, the main reason for the small sample size was the strict matching criteria to obtain comparable groups and even in this small cohort the results were not only significantly different, but also differences between groups were of clinical relevance. Matching according to birth weight was performed to rule out any potential influence of fetal growth restriction due to smoking on cerebral oxygenation. Therefore, the matching allowed us to achieve homogeneity in birth weight between the two groups.

## Conclusion

Immediately after birth, cerebral oxygenation was compromised with lower crSO2 and higher cFTOE values in stable term neonates delivered by Cesarean section from mothers who had smoked during pregnancy, compared to neonates without prenatal tobacco exposure. We assume that in the included stable term neonates, with an inconspicuous postnatal transition period, the observed lower cerebral oxygenation in the smoking group might be of questionable clinical relevance. However, our results suggest that neonates with prenatal tobacco exposure are at a higher risk of cerebral hypoxia immediately after birth. This could be even more pronounced in compromised term neonates and/or preterm neonates potentially causing cerebral injury. This observation should be implemented in the education about the harmful effects of tobacco smoking especially for women of childbearing age and those being pregnant.

## Data Availability Statement

The original contributions presented in the study are included in the article/supplementary material, further inquiries can be directed to the corresponding author/s.

## Ethics Statement

The studies involving human participants were reviewed and approved by Regional Committee on Biomedical Research Ethics, Medical University of Graz. Written informed consent to participate in this study was provided by the participants' legal guardian/next of kin.

## Author Contributions

CW and GP conceived the research idea and finalized the methods. CW, GP, and AA analyzed the data and CW wrote the first draft. CW, MB, BS, LM, EP, NH, BU, and GP contributed to data collection, interpretation of the results, and drafting and finalizing the manuscript. All authors contributed to the article and approved the submitted version.

## Conflict of Interest

The authors declare that the research was conducted in the absence of any commercial or financial relationships that could be construed as a potential conflict of interest.

## Publisher's Note

All claims expressed in this article are solely those of the authors and do not necessarily represent those of their affiliated organizations, or those of the publisher, the editors and the reviewers. Any product that may be evaluated in this article, or claim that may be made by its manufacturer, is not guaranteed or endorsed by the publisher.
